# Detection of alternative lengthening of telomeres mechanism on tumor sections

**DOI:** 10.1186/s43556-021-00055-y

**Published:** 2021-10-20

**Authors:** Eloïse Claude, Guillaume de Lhoneux, Christophe E. Pierreux, Etienne Marbaix, Maëlle de Ville de Goyet, Cécile Boulanger, An Van Damme, Bénédicte Brichard, Anabelle Decottignies

**Affiliations:** 1 Genetic & Epigenetic Alterations of Genomes Unit, de Duve Institute, UCLouvain, Brussels, Belgium; 2 Cell Unit, de Duve Institute, UCLouvain, Brussels, Belgium; 3 Department of Anatomopathology, Cliniques Universitaires Saint-Luc, Brussels, Belgium; 4 Department of Paediatric Haematology and Oncology, Cliniques Universitaires Saint-Luc, UCLouvain, Brussels, Belgium

**Keywords:** Telomere, Alternative lengthening of telomeres, Native telomeric FISH, Paediatric tumor, ALT xenograft

## Abstract

The vast majority of adult cancer cells achieve cellular immortality by activating a telomere maintenance mechanism (TMM). While this is mostly achieved by the de-silencing of h*TERT* telomerase gene expression, an alternative homologous recombination-based and telomerase-independent mechanism, known as ALT (Alternative Lengthening of Telomeres), is frequently activated in a subset of tumors, including paediatric cancers. Being absent from normal cells, the ALT mechanism offers interesting perspectives for new targeted cancer therapies. To date, however, the development of better translationally applicable tools for ALT detection in tumor sections is still needed. Here, using a newly derived ALT-positive cancer cell mouse xenograft model, we extensively examined how the previously known ALT markers could be used as reliable tools for ALT diagnosis in tumor sections. We found that, together with the detection of ultra-bright telomeric signals (UBS), an ALT hallmark, native telomeric FISH, that detects single-stranded C-rich telomeric DNA, provides a very sensitive and robust tool for ALT diagnosis in tissues. We applied these assays to paediatric tumor samples and readily identified three ALT-positive tumors for which the TMM was confirmed by the gold-standard C-circle amplification assay. Although the latter offers a robust assay for ALT detection in the context of research laboratories, it is more difficult to set up in histopathological laboratories and could therefore be conveniently replaced by the combination of UBS detection and native telomeric FISH.

**Supplementary Information:**

The online version contains supplementary material available at 10.1186/s43556-021-00055-y.

## Introduction

The ability to maintain telomere length over successive cell divisions characterizes most cancer cells and confers the dangerous replicative immortality potential that underlies indefinite cancer cell proliferation and metastasis formation [1]. In this regard, the telomere maintenance mechanism (TMM) of cancer cells rapidly emerged as a promising therapeutic target for cancer treatment. A first obstacle to this idea came after the observation that not all cancer cells use the same TMM to maintain their telomeres. In the vast majority of adult cancers, TMM activation is achieved by the de-silencing of h*TERT* telomerase gene expression (Telomerase-positive or TEL^+^ cancers). However, an alternative homologous recombination-based and telomerase-independent mechanism, known as Alternative Lengthening of Telomeres (ALT), is frequently activated in rarer cancer types, including sarcomas, astrocytomas, glioblastomas or neuroendocrine pancreatic cancers [2]. An estimated 5% of all adult cancers rely on ALT for telomere maintenance (ALT-positive or ALT^+^ cancers). Importantly, however, as the spectrum of paediatric tumors differs from the one in adults, this alternative mechanism may be used by about one out of three solid paediatric tumors, including about one-third of neuroblastoma paediatric tumors [2]. There are also evidences that some tumors may be heterogeneous and display both TMM, albeit unlikely within the same cell. Together, this has led to the second idea of developing -and possibly combining- drugs against both TMM to efficiently target the replicative potential of cancer cells [3]. So far, only one specific drug against telomerase, Imetelstat, has entered phase 2/3 clinical trials for myelofibrosis and other myeloid malignancies, after pilot study completion [4, 5]. To date, however, no specific anti-ALT drug has been identified. This results mainly from the fact that ALT relies on homologous recombination events between telomeric DNA sequences and seems to operate mostly through a bifurcated break-induced replication pathway [6] that, in its enzymatic requirements, does not differ from the DNA repair pathways that are used by the cells in response to DNA damage. If targeting homologous recombination does not offer specific therapeutic perspectives for ALT^+^ cancer treatment, the discovery that disrupting the interaction between TSPYL5 and USP7 seems to specifically induce ALT^+^ cell death offers alternative perspectives [7, 8].

A global picture of the cellular events leading to ALT activation is still missing but an increasing number of ALT-related studies performed over the last two decades increased the knowledge of the genetic and epigenetic deregulations associated with this TMM. This contributed not only to the understanding of the cellular pathways involved in ALT, but also to the identification of a series of markers that are used to detect the ALT phenotype in cultured cells or on tumor tissue sections [2]. Among them, the detection of co-localization events between telomeres and PML (Promyelocytic Leukaemia) bodies in the so-called ALT-associated PML bodies (APBs) relies on the observation, by confocal fluorescent microscopy, of PML and telomeres. However, although APBs are abundant and readily detected in cultured ALT^+^ cells, the identification of ALT^+^ tumors based on the APB criteria is more tedious and time-consuming, as ALT^+^ tumors are defined by the presence of ≥1 APB in ≥0.5% of tumor cells and require the examination of at least 2000 nuclei [9]. Several cases of ALT^+^ cells lacking APBs were however reported in the literature [10–12] and the co-localization of telomeres with PML bodies is therefore not considered an absolute requirement for classification as ALT^+^ [13]. A second ALT marker is based on the heterogeneous telomere length profile of ALT^+^ cells that can be evaluated by either telomeric Fluorescent in situ Hybridization (FISH) or immunofluorescence (IF) against one of the telomere-binding proteins of the shelterin complex. Telomere length heterogeneity, however, is not always easy to identify as the clustering of telomeres that happens in normal cells may result in an apparent heterogeneity of telomere length. In addition to telomere length heterogeneity, some ALT^+^ cancer cells display ultra-bright telomeric foci that, when arising, are easily detected by FISH and allows a clear discrimination with normal cells [13]. Whether or not all ALT^+^ cancer cells display such ultra-bright telomeric foci is however not known. Detection of a third marker of ALT, namely the presence of extrachromosomal and partially single-stranded C-rich telomeric circles, relies on an enzymatic in vitro assay called the CCA (C-circle assay). This robust assay, first reported in 2009 [14], has become increasingly popular in the ALT field and is now universally recognized as valuable ALT marker. The CCA, however, is less convenient for diagnostic purposes in routine as it requires *i*) larger amounts of tumor tissue, *ii*) genomic DNA extraction and *iii*) an in vitro enzymatic reaction assay followed by either a dot/slot-blot analysis using a telomeric probe or a qPCR analysis [15, 16], even though the latter is less sensitive than the blot.

We recently reviewed the assays that are currently used to detect ALT or TEL activation in tumor tissues and we underlined the paucity of reliable diagnostic tools compatible with translational applications [2]. Very recently, the question of non-invasive tools for TMM assessment in brain tumor patients was investigated. As proof-of-concept, authors showed that, in orthotopic tumor xenograft models, TEL^+^ and ALT^+^ tumors could be discriminated through combinations of ^1^H- and hyperpolarized ^13^C-magnetic resonance spectroscopy-detectable metabolic signatures, opening interesting perspectives for the future non-invasive diagnosis of patients with low-grade oligodendrogliomas or low-grade astrocytomas [17]. Whether this non-invasive technique would be transposable to other tumor types still needs to be evaluated. While non-invasive techniques are undeniably interesting to, for instance, follow-up the response to anticancer treatments, most of the time, a biopsy is performed when the tumor is detected and tumor material is thus available for the initial TMM diagnosis.

Here, we addressed the possibility of developing new tools for ALT detection on tumor biopsies that may be compatible with clinical biology routine. To this end, we first developed a new ALT^+^ tumor xenograft model to re-evaluate a series of previously described ALT detection assays on tumor tissues. In a second step, we developed a new sensitive assay for ALT detection on tissue sections based on a native FISH protocol that detects single-stranded C-rich telomeric DNA (ss-TeloC), one hallmark of ALT^+^ cells [18]. Finally, we applied these various tests to a collection of solid paediatric tumor sections and identified ALT^+^ osteosarcoma and neuroblastoma tumors.

## Results

### LB857/ALT^+^ myxoid sarcoma cells readily form macroscopic tumors in immunodeficient NSG mice

To date, conflicting results have been obtained regarding the availability of reliable ALT^+^ tumor xenograft models. While tumors poorly developed 3 months after injection of U2OS/ALT^+^ osteosarcoma cells in immuno-compromised nude mice [19], the same cells very inefficiently formed small tumors 6 months after injection in immunodeficient NOD scid gamma (NSG) mice [20]. Similarly, while tumor formation was not observed in nude mice injected with the SaOS-2/ALT^+^ cell line [19], the same cells were able to form macroscopic tumors in NSG mice after a long incubation time of 3 months [20]. Hence, none of the two most frequently used ALT^+^ sarcoma cell lines, U2OS/ALT^+^ or SaOS-2/ALT^+^, appears to provide a robust ALT^+^ tumor xenograft model. This observation may however not be generalized to all ALT^+^ cell lines as the study by Lauvrak et al [20] reported efficient tumor formation in NSG mice injected with other ALT^+^ osteosarcoma cell lines, including CAL-72, ZK-58, KPD or G-292, although, here too, conflicting results were observed as the ZK-58 and KPD cell lines failed to develop tumors in nude mice [19].

**Fig. 1:**
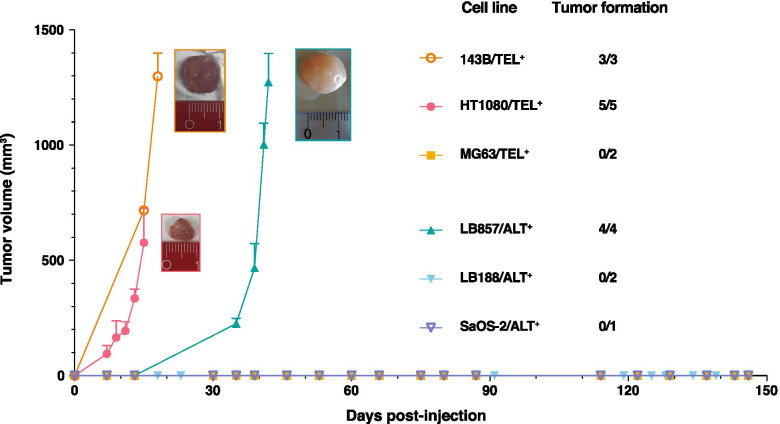
Establishment of an ALT^+^ tumor xenograft model in NSG mice. Tumor volume was monitored over time with a digital caliper after injection of 10^6^ cells in the flank of NSG mice. 143B//TEL^+^ is an osteosarcoma cell line (*n* = 3); HT1080/TEL^+^ comes from a fibrosarcoma (*n* = 5); LB857/ALT^+^ is a myxoid sarcoma cell line (*n* = 4); MG63/TEL^+^ and LB188/ALT^+^ derive from, respectively, an osteosarcoma and a rhabdomyosarcoma (*n* = 2) tumor and SaOS/ALT^+^ is an osteosarcoma cell line (*n* = 1). Mean + SEM. Representative pictures of the xenografts are shown

To identify cancer cells that would provide a robust ALT^+^ tumor xenograft model, we compared the ability of various ALT^+^ cancer cell lines to form macroscopic tumors in immunodeficient NSG mice within a time frame compatible with possible future drug testing experiments. We selected the commercially-available osteosarcoma cell line SaOS-2/ALT^+^ and two in-house derived sarcoma cell lines: the LB188/ALT^+^ rhabdomyosarcoma cell line [21] and the LB857/ALT^+^ myxoid sarcoma cell line [22]. We also included commercially available TEL^+^ cancer cell lines as controls: two osteosarcoma cell lines (MG63 and 143B) and one fibrosarcoma cell line (HT1080). As expected from previous reports [19, 20, 23], both 143B/TEL^+^ and HT1080/TEL^+^ cell lines readily formed macroscopic tumors as soon as 1–2 week(s) after injection, while MG63/TEL^+^ cells had not developed into palpable tumors when the experiment was stopped 5 months post-injection (Fig.1). In agreement with previous results obtained in nude mice [19], the injected SaOS-2/ALT^+^ cells did not develop into macroscopic tumors within 5 months after injection (Fig.1). Similarly, the in-house established LB188/ALT^+^ rhabdomyosarcoma cell line had still not formed tumors when mice were sacrificed 5 months after injection (Fig.1). Interestingly however, we found that LB857/ALT^+^ myxoid sarcoma cells consistently and rapidly developed into macroscopic tumors 1 month after their injection in NSG mice (4/4) (Fig.1). Together, we identified the LB857/ALT^+^ myxoid sarcoma cell line as interesting candidate for robust ALT^+^ tumor xenograft model.

In a next step, we wanted to take advantage of the LB857/ALT^+^ tumor xenograft model to set up tools for ALT marker screening on tumor tissue sections, coming from either cryopreserved or Formalin-Fixed Paraffin-Embedded (FFPE) samples. We selected the HT1080/TEL^+^ xenograft as negative control for ALT marker detection.

### Validation of ALT markers in LB857/ALT^+^ and HT1080/TEL^+^ cell lines

**Fig. 2:**
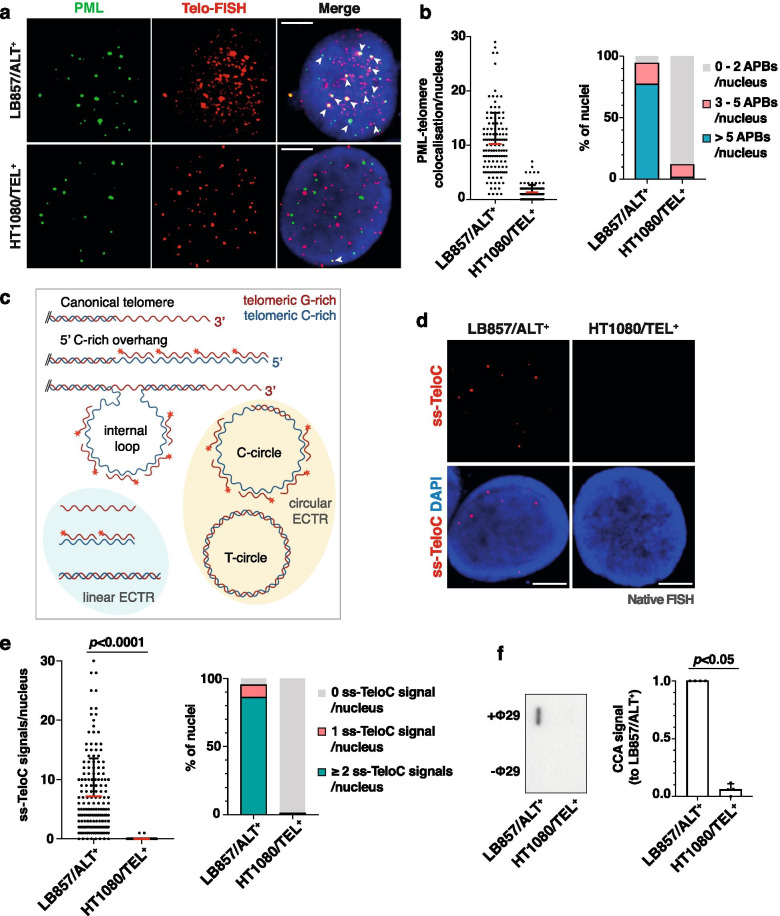
ALT marker detection in LB857/ALT^+^ and HT1080/TEL^+^ cancer cell lines. **a** Representative images of PML (IF, green) and telomeres (denaturing FISH, red) staining. Arrowheads show colocalization events. Scale bars: 5 μm. **b** Quantification of the number of colocalization events between PML and telomeres per nucleus, with each dot representing one nucleus. The red line marks the mean value and SD bar is shown. The graph on the extreme right represents the frequency of nuclei presenting 0 to 2 (grey), 3 to 5 (pink) or more than 5 (blue) PML-telomere colocalization event(s) per nucleus. Single experiment in which at least 100 nuclei were quantified. **c** Schematic of the native FISH assay (created with BioRender.com). The various types of ss-TeloC sequences found in ALT^+^ cells are recognized by the fluorescently-labelled G-rich probe (indicated with a red star) in non-denaturing conditions. **d** Representative images for native FISH signals in LB857/ALT^+^ and HT1080/TEL^+^ cancer cells. ECTR: Extrachromosomal Telomeric Sequence. **e** Quantification of native FISH signals. Each dot represents the number of ss-TeloC signals in one nucleus. The red line marks the mean value and SD bar is shown. The frequency of nuclei showing 0 (grey), 1 (pink) or more than 1 (green) ss-TeloC signal per nucleus is shown on the stacked column chart. Two independent experiments were performed in which at least 80 nuclei were quantified. Scale bars: 5 μm. **f** Slot-blot of the C-circle assay with (+Φ29) or without (−Φ29) Phi29 DNA polymerase. Quantification of the slot-blot on the right shows the mean + SD (*n* = 4). Statistical analyses were performed using the nonparametric Mann-Whitney test

Before analyzing xenografts, we validated the assays on the corresponding cultured cells. Three distinct types of staining were performed to detect, respectively, APBs (denaturing telomeric FISH combined with anti-PML IF), telomere length heterogeneity (denaturing telomeric FISH) and single-stranded C-rich telomeric DNA (native telomeric FISH). As expected, LB857/ALT^+^ cell line was positive for APB and had heterogeneous telomere length (Fig.2a and b). Ss-TeloC sequences were also detected in LB857/ALT^+^ cells (Fig.2c-e) and in other ALT^+^ cell lines (Online Resource 1) as recently shown by others [18]. Conversely, co-localization events between telomeres and PML bodies were drastically lower in the HT1080/TEL^+^ cell line (Fig.2a and b), and telomere length, assessed by denaturing FISH, appeared homogeneous (Fig.2d and e). More strikingly, native FISH signals were not detected in 158 out of 160 HT1080/TEL^+^ nuclei that we analyzed and the two positive nuclei showed no more than one signal (Fig.2e). C-circle presence was also assessed using the previously described CCA [24]. Not surprisingly, C-circle amplification in LB857/ALT^+^ cells was significantly higher than in HT1080/TEL^+^ cells (Fig.2f; *p* = 0.03), confirming the reliability of this assay.

### Microscopy-based ALT marker investigation in LB857/ALT^+^ and HT1080/TEL^+^ tumor xenografts: APBs, denaturing and native telomeric FISH

**Fig. 3:**
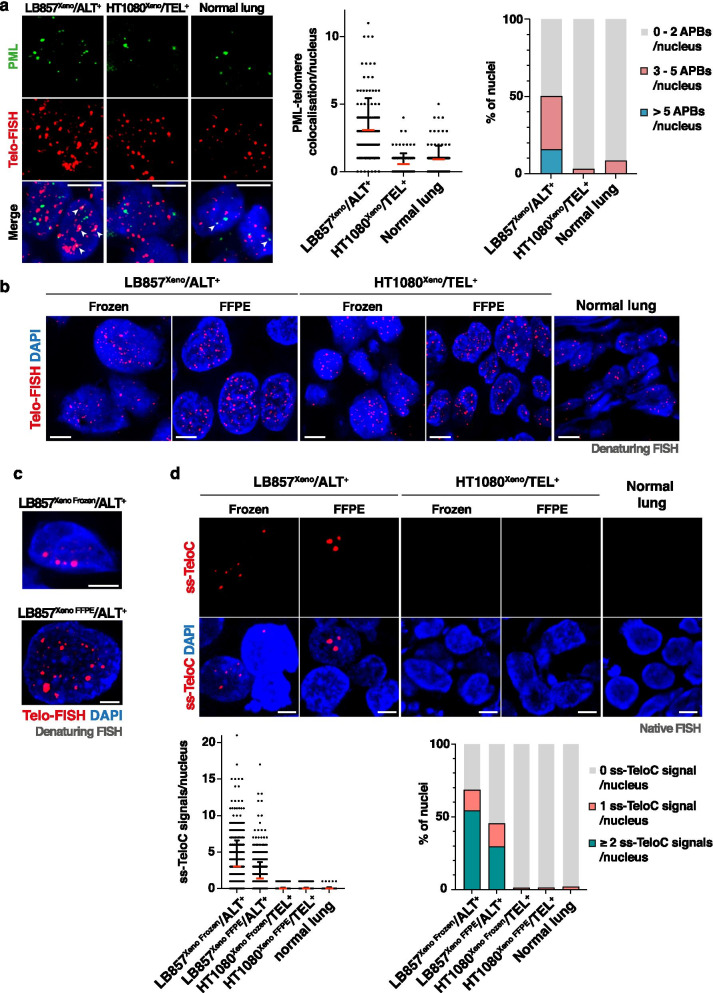
Native telomeric FISH for single-stranded C-rich telomeric DNA visualisation as a potent tool for ALT detection in xenograft-derived tumor tissues. **a** Representative images of PML (IF, green) and telomeres (denaturing FISH, red) staining on frozen xenografts, obtained with LB857/ALT^+^ or HT1080/TEL^+^ cell lines, and healthy human lung sections. Arrowheads show colocalization events. Scale bars: 5 μm. Quantification of PML-Telomeres colocalization events per nucleus is shown on the right, with each dot representing one nucleus. The red line marks the mean value and SD bar is shown. The graph on the extreme right represents the frequency of nuclei presenting 0 to 2 (grey), 3 to 5 (pink) or more than 5 (blue) colocalization events per nucleus. Single experiment in which at least 100 nuclei were quantified. **b** Representative images for denaturing telomeric FISH in sections from either FFPE or frozen LB857^Xeno^/ALT^+^ and HT1080^Xeno^/TEL^+^ xenografts, and frozen healthy human tissue. Scale bars: 5 μm. **c** Representative images for ultra-bright telomeric signals identified in FFPE and frozen LB857^Xeno^/ALT^+^ sections. Scale bars: 5 μm. **d** Representative images for native FISH experiment in the tissues described in **b**. The quantification is shown below, with each dot representing the number of ss-TeloC signals in one nucleus. Mean + SD. The frequency of nuclei showing 0 (grey), 1 (pink) or more than 1 (green) ss-TeloC signals per nucleus is shown in the graph on the right. Single experiment in which at least 200 nuclei were quantified. Scale bars: 5 μm

We next tested the stainings on LB857/ALT^+^ and HT1080/TEL^+^ xenograft cryosections. Although the overall frequency was lower than in the corresponding cell lines, PML-telomere co-localization events were more prevalent in LB857/ALT^+^ than in HT1080/TEL^+^ xenograft sections (Figs. 2b and 3a). As control, PML-telomere co-localization was also assessed in human healthy lung cryosections and some co-localization events were detected as well, consistent with previous report in normal cells [25] (Fig .3a). Hence, the APB assay did not provide a black and white answer for the evaluation of ALT phenotype. Moreover, we could not get a suitable PML staining in FFPE samples. Similarly, the evaluation of telomere length heterogeneity by FISH to discriminate between ALT^+^ and ALT^−^ tissue samples turned out to be challenging since both HT1080/TEL^+^ xenograft and normal lung tissue were, to some extent, displaying heterogeneous signals (Fig. 3b). Conversely, ultra-bright telomeric FISH signals (UBS) could only be detected in ALT^+^ xenografts and were never detected in TEL^+^ xenograft samples (Fig. 3c, Table 1). The frequency of UBS^+^ nuclei was however low, amounting to, respectively, 0.9% and 1.5% of the nuclei in frozen and FFPE xenograft samples (Table 1).

**Table 1:**
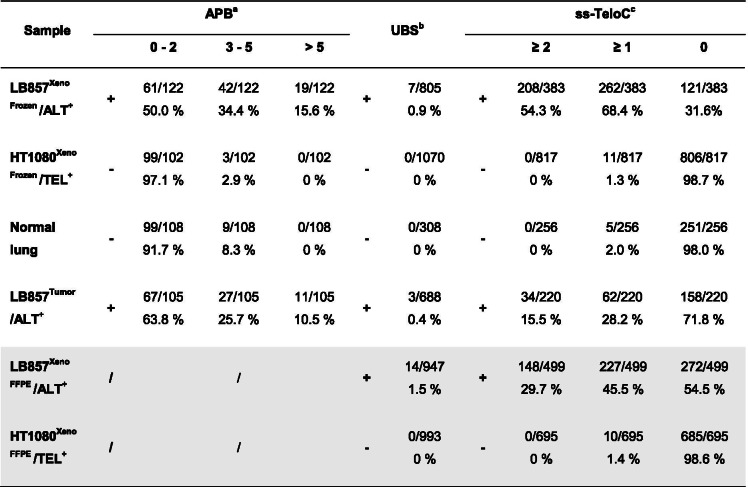
APBs, UBS and ss-TeloC detection in xenografts, normal human lung and an ALT+ tumor

FFPE samples are highlighted in grey**.** ^a^ Positive if ≥5% of nuclei have > 5 APBs; Negative if ≥80% of nuclei have < 3 APBs. ^b^ Positive if ≥0.2% of nuclei with at least one telo-FISH signal of ≥2500 RFU; Negative if no nuclei with telo-FISH signal ≥2500 RFU. ^c^ Positive if ≥10% of the nuclei with ≥1 ss-TeloC signal; Negative if < 5% of nuclei with ≥1 ss-TeloC signal. APB: ALT-associated PML Body; UBS: Ultra-bright telomere FISH signal; ss-TeloC: single-stranded C-rich telomeric DNA; Relative Fluorescence Unit

To increase the robustness of microscopy-based assays to discriminate between ALT^+^ and TEL^+^ phenotypes, especially in FFPE samples, we next assessed whether native telomeric FISH could have any added value. To our knowledge, this assay had never been used to detect the ALT mechanism in tissue sections. As shown in Fig. 3d, ss-TeloC could be detected by native FISH in ALT^+^ xenografts, providing a very discriminant tool to distinguish between ALT^+^ and TEL^+^, both in frozen and FFPE samples. Indeed, nuclei displaying more than one signal were not detected in HT1080/TEL^+^ xenograft samples nor in normal lung sections while at least 30% of nuclei had ≥2 ss-TeloC foci in the LB857/ALT^+^ xenograft samples (Fig. 3d, Table 1).

Altogether, the above data suggest that native telomeric FISH provides a robust and convenient assay for ALT detection in both frozen and FFPE tumor tissues. Using a threshold of ≥1 ss-TeloC signal per nucleus on a total analysis of about 200 nuclei, at least 45% of LB857/ALT^+^ xenograft nuclei were positive for the marker while only 1.4% of HT1080/TEL^+^ xenograft nuclei were ss-TeloC^+^ (Table 1).

### C-circle analysis provides another stringent assay for ALT^+^ tumors

**Fig. 4:**
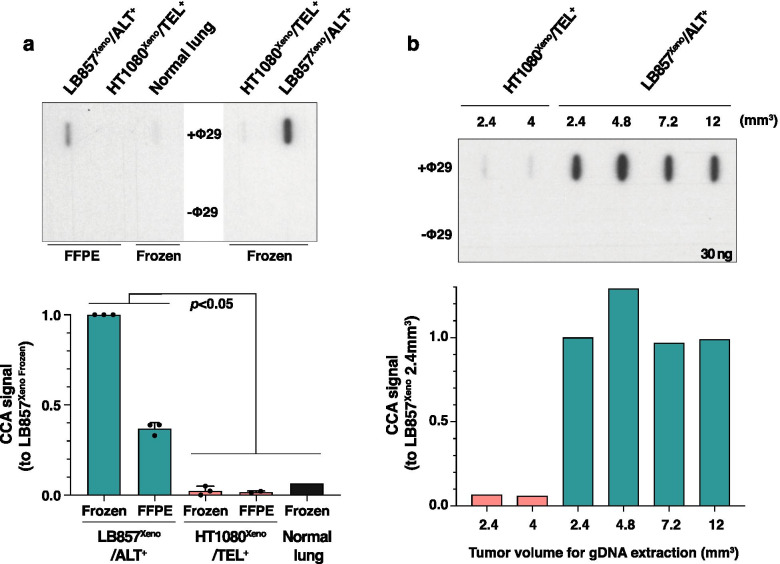
C-circle detection from tumor tissue sections as another reliable ALT marker. **a** Slot-blot of the C-circle assay with (+Φ29) or without (−Φ29) Phi29 DNA polymerase performed on gDNA extracted from sections of either FFPE or frozen ALT^+^ (green) and TEL^+^ (pink) xenografts as indicated. Frozen human healthy lung tissue was used as control. Quantification of the slot-blot below shows mean + SD. A single experiment was performed on the frozen lung tissue while two to three independent experiments were performed on the xenograft tissues. Statistical analysis was performed using the nonparametric Mann-Whitney test to compare ALT^+^ and ALT^−^ groups. **b** Slot-blot of the C-circle assay with (+Φ29) or without (−Φ29) Phi29 DNA polymerase performed with 30 ng of gDNA extracted from different volumes of frozen LB857^Xeno^/ALT^+^ (green) or HT1080^Xeno^/TEL^+^ (pink) xenograft tissues as indicated. Quantification is shown below (*n* = 1)

As mentioned above, the CCA for ALT detection is less convenient for translational application. Since the assay is robust and widely used in research laboratories, we nevertheless wanted to compare its efficiency on frozen and FFPE tumor sections. As expected, CCA worked on both frozen and FFPE xenograft samples and gave, like in cell lines (Fig. 2f), a significant difference between ALT^+^ and ALT^−^ samples (Fig. 4a; *p* = 0.03). Note that the frozen LB857^Xeno^/ALT^+^ sample gave a stronger CCA signal than the FFPE sample and that some background signal was detected with the frozen TEL^+^ xenograft (Fig. 4a). We also investigated the minimal volume of tissue needed to enable C-circle detection. We found that 2.4 mm^3^ of cryopreserved LB857/ALT^+^ xenograft were enough to perform the CCA in our experimental conditions (Fig. 4b).

### Application of the ALT detection tools to paediatric tumor samples identifies new ALT^+^ tumors

**Fig. 5:**
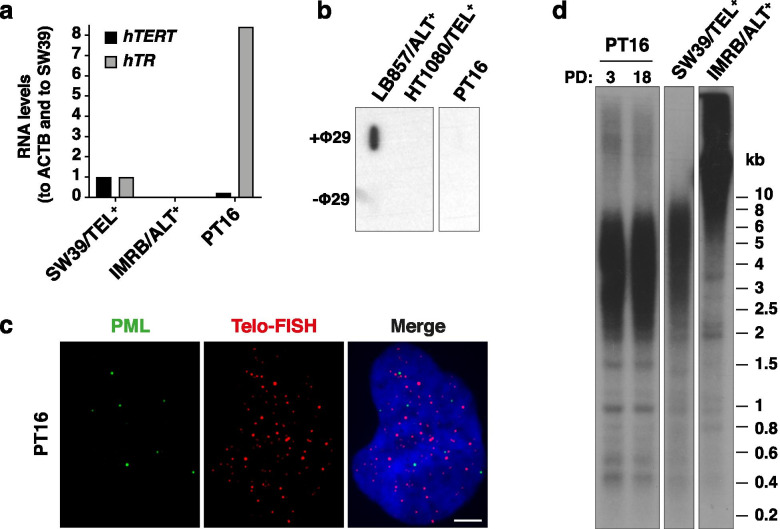
Characterization of TMM in PT16-derived cell line. **a** *hTERT* (black) and *hTR* (grey) RNA levels, normalized to *ACTB* and to SW39/TEL^+^ immortalized cell line, in PT16 cell line and IMRB/ALT^+^ immortalized cell lines (*n* = 1). **b** Slot-blot of the C-circle assay with (+Φ29) or without (−Φ29) Phi29 DNA polymerase in PT16 cell line compared to LB857/ALT^+^ and HT1080/TEL^+^ cell lines. **c** Representative images of PML (IF, green) and telomeres (denaturing FISH, red) staining in PT16 cell line. Scale bar: 5 μm. **d** TRF analysis of telomere length of PT16 cell line at population doubling (PD) 3 or 18 compared to SW39/TEL^+^ and IMRB/ALT^+^

In a next step, we wanted to apply the ALT detection tools to sections from 13 paediatric tumor samples collected over the last 2 years. These included a total of 10 tumor types (primary tumors, tumor relapses or metastases) that are listed in Table 2. For one of these tumors, coming from a paediatric high-grade sarcoma (PT16^Tumor^), a cell line has been successfully derived that we classified as TEL^+^ based on h*TERT* and h*TR* expression (Fig. 5a), lack of C-circle production (Fig. 5b), absence of APBs (Fig. 5c), and telomere length homogeneity (Fig. 5d). The corresponding tissue sample has therefore been used as TEL^+^ control. As ALT^+^ control, we used the metastasis from which the LB857/ALT^+^ cell line had been derived (adult myxoid sarcoma). To distinguish between the tumor and the cell line for PT16 and LB857 samples, we used a distinct terminology for tumor samples: PT16^Tumor^/TEL^+^ and LB857^Tumor^/ALT^+^.

**Table 2:**
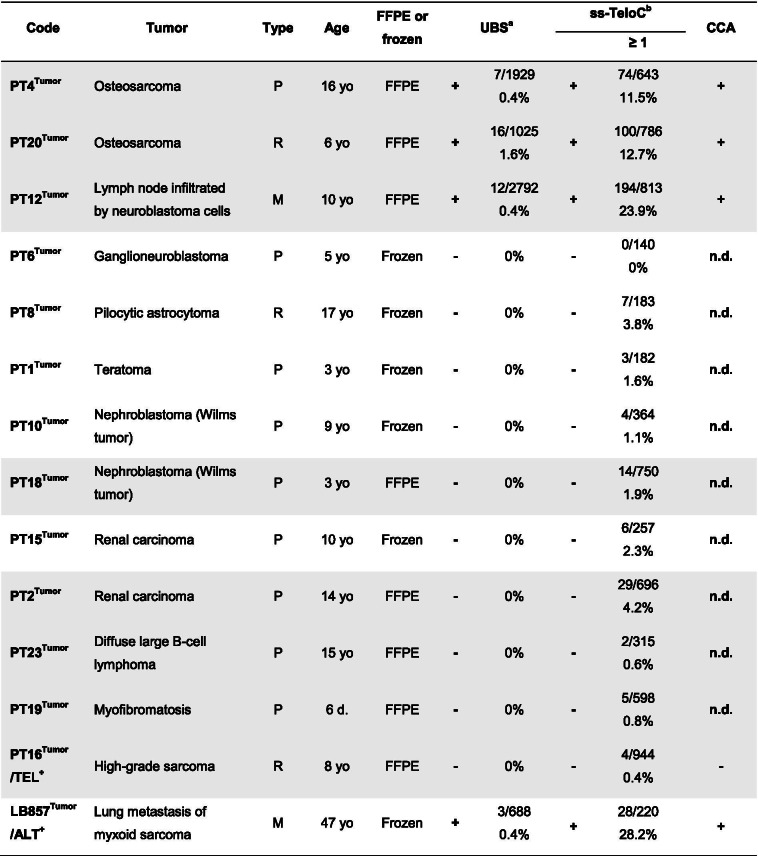
Overview of the tumors included in this study and the results for the different markers assessed

FFPE samples are highlighted in grey. ^a^ Positive if ≥0.2% of nuclei with at least one telo-FISH signal of ≥2500 RFU; Negative if no telo-FISH signal of ≥2500 RFU detected in at least 1000 nuclei; ^c^ Positive if ≥10% of the nuclei with ≥1 ss-TeloC signal; Negative if < 5% of nuclei with ≥1 ss-TeloC signal. APB: ALT-associated PML Body; UBS: Ultra-bright telomere FISH signal; ss-TeloC: single-stranded C-rich telomeric DNA; CCA: C-circle Assay; Relative Fluorescence Unit; n.d.: not determined

**Fig. 6:**
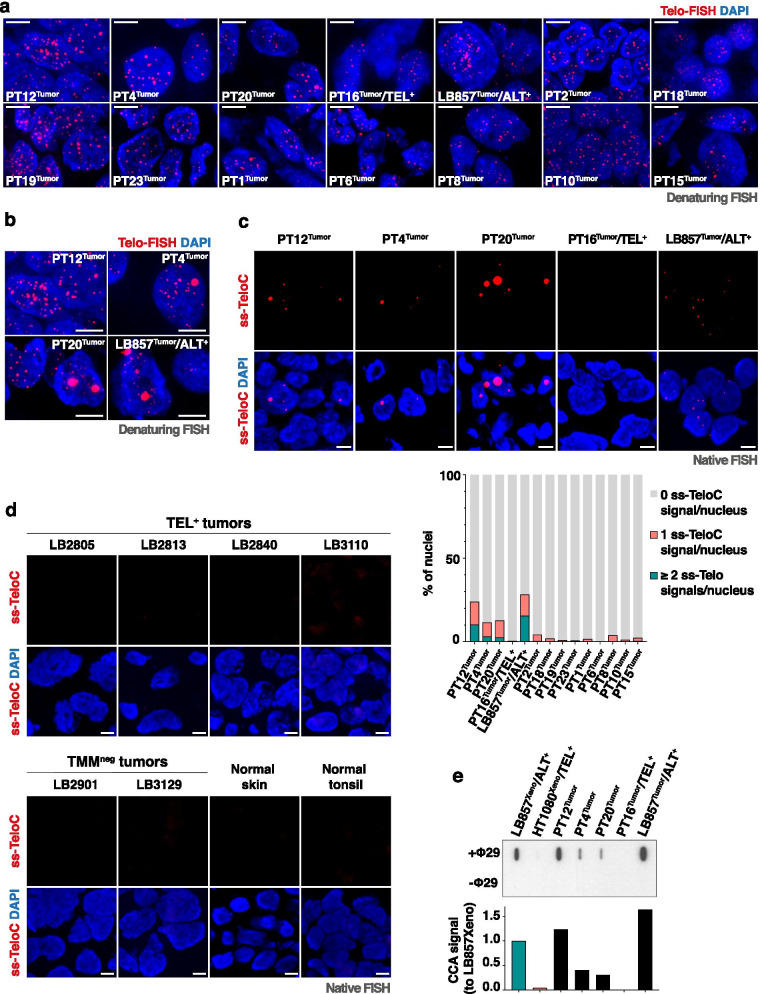
ALT detection in paediatric tumor samples. **a** Representative images for denaturing telomeric FISH in sections from paediatric tumors and adult LB857^Tumor^/ALT^+^ section. Scale bars: 5 μm. **b** Representative images for ultra-bright telomeric signals identified in the paediatric PT12^Tumor^, PT4^Tumor^, PT20^Tumor^ tumor sections and in the LB857^Tumor^ section from adult ALT^+^ tumor. Scale bars: 5 μm. **c** Representative images of native FISH signals in the same tissues as in **b**, as well as in the PT16^Tumor^/TEL^+^ tumor section used as negative control. The quantification of the native FISH experiment performed in all the paediatric tumors is shown below as frequency of nuclei scored as 0 (grey), 1 (pink) or more than 1 (green) ss-TeloC signals per nucleus. Single experiment in which at least 140 nuclei were quantified. Scale bars: 5 μm. **d** Representative images of native FISH signals in sections from melanoma tumors with either active telomerase (TEL^+^ tumors: LB2805, LB2813, LB2840, LB3110) or no detectable TMM (TMM^neg^ tumors: LB2901, LB3129) [27]. Native FISH assay was also performed on sections from normal human skin or tonsils. Scale bars: 5 μm. **e** Slot-blot of the C-circle assay with (+Φ29) or without (−Φ29) Phi29 DNA polymerase performed on FFPE PT12^Tumor^, PT4^Tumor^, PT20^Tumor^ and frozen LB857^Tumor^ samples, as well as FFPE PT16^Tumor^/TEL^+^ used as negative control. We also included the FFPE TEL^+^ and ALT^+^ xenograft tissues as, respectively, negative and positive controls. Quantification is shown below (*n* = 1)

We first looked at telomere length heterogeneity and ultra-bright telomeric signals using denaturing telomeric FISH. For most tumor samples, telomere length heterogeneity was not easily assessed (Fig. 6a). However, we detected a low frequency of nuclei displaying ultra-bright telomeric signals in three tumor sections, including one neuroblastoma (PT12^Tumor^) and two osteosarcomas (PT4^Tumor^ and PT20^Tumor^) with, respectively, 0.4%, 0.4% and 1.6% of UBS^+^ nuclei (Fig. 6b and Table 2). We next screened the paediatric tumors for the presence of ss-TeloC signals by native FISH. Using an arbitrary threshold of more than 10% of the analysed nuclei displaying at least one ss-TeloC signal, we found that the three UBS^+^ tumor samples were also positive for that second marker, while the remaining samples were negative using the same criteria (Fig. 6c and Table 2). Additional negative controls for the native telomeric FISH assay were performed on sections from melanoma tumors that we previously characterized as either expressing telomerase (LB2805, LB2813, LB2840, LB3110) or not having any telomere maintenance mechanism (LB2901, LB3129) [26] (Fig. 6d). Sections from skin and tonsils were also included as additional controls for normal human tissues (Fig. 6d).

To confirm the activation of ALT in these three ss-TeloC^+^ paediatric tumor samples, we performed the CCA on genomic DNA extracted from the tumors and found that all three samples were indeed positive for C-circles, while the PT16 tumor, with activated telomerase, was not (Fig. 6e). In the tested samples, the intensity of CCA signals appeared to correlate better with the abundance than with the intensity of ss-TeloC foci (Fig. 6c and e). Collectively, these results suggest that native telomeric FISH offers a powerful tool for ALT detection in tumor samples. More paediatric/adult tumor samples should however be tested in the near future to further validate the suitability of the native FISH assay for ALT detection.

## Discussion

With the expected development of TMM-specific anticancer drugs in the near future, the need to identify reliable and translationally applicable tools for ALT detection in tumor sections has progressively increased over the last years. Paediatric cancers, because of *i*) the higher prevalence of ALT and *ii*) the urgent need to develop targeted therapies, may notably benefit from such tools. Paediatric cancers indeed dramatically suffer from the lack of new less toxic therapies and the ALT mechanism, being absent from normal cells, offers interesting perspectives in this regard [8].

To re-evaluate the currently available tools for ALT screening, and to develop new assays, on either FFPE or frozen tumor sections, we first set up the LB857/ALT^+^-derived mouse xenograft model. We found that single-stranded C-rich telomeric DNA visualization, using native FISH, provides a sensitive and convenient assay for ALT detection that is compatible with routine FISH-based tests in histopathological laboratories. At least 2 native telomere FISH signals were detected in 30% of the examined tumor cells in the LB857/ALT^+^-derived FFPE xenograft and even more in the frozen sample, whereas none of the nuclei had ≥2 native FISH signals in either the HT1080/TEL^+^ xenograft or normal lung tissue (Table 1). In our hands, this assay gave a clearer black and white answer than the denaturing telomeric FISH for which the assessment of telomere length heterogeneity was not always straightforward. As for ultra-bright telomeric signals, when detected, their frequency was very low, amounting to less than 2% of the nuclei. Our data further suggest that ultra-bright telomere detection could potentially be missed in a primary tumor, especially if the percentage of tumor cells is not high. Here, we also confirmed the reliability of the CCA first reported more than 10 years ago [14]. Initially developed on genomic DNA extracted from ALT^+^ cell lines, the technique turned out to be sensitive enough to detect C-circles in genomic DNA extracted from ALT^+^ malignant gliomas in a large-scale study including 63 tumors [27]. That study further suggested that the CCA was more straightforward than the screen for APBs in ALT detection [27]. Here, we reached similar conclusions and also detected co-localization events between telomeres and PML in healthy human lung tissue, thus reducing the confidence in the APB assay to identify the ALT phenotype on tumor sections. Together with the observation that, on one hand, the co-localization of telomeres with PML is not always observed in ALT^+^ tumors [13, 28] and, on the other hand, the ALT phenotype is not always confirmed in tumors defined as APB^+^ [29], we believe that the screen for APBs is not a robust assay for ALT tumor detection. In this regard, it is also important to remind that the identification of ALT^+^ tumors based on the APB criteria is extremely time-consuming, making it incompatible with translational analyses [9]. Altogether, we therefore propose that a combination of UBS detection, by denaturing telomeric FISH, and single-stranded C-rich telomeric DNA visualization, using native FISH, may provide a reliable ALT diagnostic tool in tumor samples.

In this study, we searched for ALT markers in 13 solid paediatric tumors by using a combination of telomeric FISH for UBS detection, native telomeric FISH and CCA. We identified three tumors positive for all three ALT markers: one neuroblastoma and two osteosarcomas. These results are in line with the previously reported high prevalence of ALT phenotype in osteosarcoma and paediatric neuroblastoma [2]. In the future, additional tumor samples should be screened using the native FISH assay to confirm its robustness as ALT detection tool.

## Materials and methods

### Cell culture

Cancer cell lines expressing telomerase are labelled as TEL^+^ while cells with an ALTernative mechanism for telomere maintenance are indicated as ALT^+^. The osteosarcoma cell lines, SaOS-2/ALT^+^ (ATCC, HTB-85), U2OS/ALT^+^ (ATCC, HTB-96), MG63/TEL^+^ (ATCC, CRL-1427) and 143B/TEL^+^ (Coriell Institute for medical Research, New Jersey, USA) were grown in DMEM (Gibco) supplemented with 10% FBS (Gibco), 1% glutamine (Gibco) and 1% penicillin/streptomycin (PS) (Gibco). The HT1080/TEL^+^ fibrosarcoma cell line (kindly provided by J. Lingner, EPFL, Lausanne), the IMRB/ALT^+^ (Coriell Institute for medical Research, New Jersey, USA), SW39/TEL^+^ (kindly provided by W. Wright, UT Southwestern, Dallas, USA), SI27/TEL^+^, SI24/ALT^+^ [30] and VA13/ALT^+^ [21] SV40T-immortalized human fetal lung fibroblasts were grown in EMEM (Gibco) supplemented with 10% FBS (Gibco), 1% NEAA (Gibco) and 1% PS (Gibco). The LB188/ALT^+^ rhabdomyosarcoma [21] and the LB857/ALT^+^ myxoid sarcoma [22] cell lines were grown in IMDM (Gibco) supplemented with 10% FBS (Gibco), 1% NEAA (Gibco) and 1% PS (Gibco). HITES (10 nM hydrocortisone, 10 mg/L insulin, 100 mg/L transferrin, 10 nM estradiol, and 30 nM sodium selenite) was added in this same medium for the culture of the PT16 sarcoma cell line that we obtained in this study. Cells were cultured at 37 °C under a humidified atmosphere of 5% CO_2_.

### Xenograft experiments

All animal studies were performed in accordance with national and institutional guidelines for animal care, under permit numbers 2016/UCL/MD/009 and 2020/UCL/MD/012. Xenograft experiments were performed as described previously [26]. Briefly, locally bred 6 to 8-week-old female NSG mice (Jackson Laboratory) were injected subcutaneously with 10^6^ tumor cells resuspended into 100 μl of 1:1 phosphate-buffered saline (PBS) and Matrigel (VWR International). Tumor size was measured with a digital caliper. Animals were sacrificed by cervical dislocation before tumors reached 1500 mm^3^ or when any kind of animal suffering was detected or if no sign of tumor formation was detected after 5 months. The resected tumors were either directly snap-frozen in isopentane chilled on dry ice or fixed in formalin for 24 h and paraffin-embedded using Tissue-Tek VIP 6-E2 Tissue Processor (Sakura).

### Tissue samples

Frozen or Formalin-Fixed Paraffin-Embedded (FFPE) sections of paediatric tumors were acquired from the Cliniques universitaires St-Luc, Brussels. Frozen sections from the LB857/ALT^+^ myxoid sarcoma tumor (lung metastasis), melanoma tumors (LB2805/TEL^+^, LB2813/TEL^+^, LB2840/TEL^+^, LB3110/TEL^+^, LB2901/TMM^neg^, LB3129/TMM^neg^) [26] and normal tonsils or skin were provided by the Ludwig Institute for Cancer Research (Brussels branch). Analysis of human normal lung tissue was also performed on the healthy surgical margin of LB857/ALT^+^ tumor section. This study has been approved by the local ethics committee (Comité d’Ethique Hospitalo-Facultaire) under reference 2017/27JUI/335 (Belgian registry: B403201732874).

### Immunofluorescence (IF), native and denaturing telomeric FISH

Five μm-thick sections were cut from the frozen tumors before fixation in acetone for 5 min, followed by 10 min in 4% formaldehyde. For FFPE samples, 6 μm-thick sections were first dewaxed through 3 successive incubations of 3 min each in xylene before rehydration through a graded ethanol series (100%, 95%, 70% and 30%) for 3 min each. For both FFPE and frozen sections, slides were then washed in PBS and microwave heated at 450 W for 10 min in citrate buffer (10 mM sodium citrate, 0.1% Tween-20, pH 6.0). This step was omitted for native FISH to avoid DNA denaturation. Slides were then cooled down, washed in PBS and incubated into permeabilization buffer (20 mM Tris-HCl pH 8.0, 50 mM NaCl, 3 mM MgCl_2_, 300 mM sucrose, 0.5% Triton X-100) for 1 h at 37 °C. For staining experiments on cultured cells, 20,000 cells were seeded onto 4-well slides in 150 μl of medium and incubated overnight at 37 °C under a humidified atmosphere of 5% CO_2_. The next day, cells were washed twice with PBS, cytoplasm was pre-extracted with permeabilization buffer prior fixation with 3.7% formaldehyde and 2% sucrose in PBS for 15 min at room temperature (RT) and finally permeabilized again 10 min at RT. All subsequent treatments were identical for slides with cells or tissue sections.

For native and denaturing FISH, slides were first treated with 100 μg/mL RNase A for 1 h at 37 °C and then serially dehydrated, 2 min each, with 70%, 85% and 100% ethanol baths, air dried, overlaid with hybridization solution (160 nM TeloG Exiqon LNA™ red probe, 50% deionized formamide, 2x Saline-Sodium Citrate (SSC), 1x Blocking reagent (Roche)) and finally incubated at 83 °C for 3 min with a coverslip on. For native FISH, slides were hybridized at RT instead of 83 °C. Slides were further incubated with the probe at RT for at least 1 h. Unbound probe was washed off successively as follows: twice 15 min in 50% formamide, 2x SSC, 20 mM Tris-HCl pH 7.4 and three times 5 min in 150 mM NaCl, 0.05% Tween-20, 50 mM Tris-HCl pH 7.4. Slides were serially dehydrated again, air dried and mounted with mounting medium (23.5 mg/ml DABCO (Sigma-Aldrich), 20 mM Tris-HCl pH 7.4, 90% v/v glycerol) containing 0.6 μg/ml DAPI. Images were acquired with the Cell Observer Spinning Disk confocal microscope (Zeiss) with 100X objective or 40X for native FISH on tissue. Pictures were analyzed using ImageJ software (National Institute of Health). Note that we maintained the same threshold for samples from the same experiment. For ultra-bright telomeric signals, slides were scored as positive if the integrated density of a foci was more than 2500 RFU (Relative Fluorescence Units) in a nucleus of the sample.

For PML IF combined with telomeric FISH, slides were re-permeabilized for 1 h at 37 °C after the second dehydration step post-FISH. Following three washes with PBS-Tween (0.1%), slides were incubated for 45 min at RT in blocking solution (1% BSA, 10% normal goat serum (Cell Signaling Technology), 0.1% Triton X-100 in PBS) before incubation overnight at 4 °C with anti-PML (1:100, Santa Cruz Biotechnology, sc-966) diluted in blocking solution. The next day, slides were washed 3 times with PBS-Tween and incubated for 40 min at 45 °C with anti-mouse IgG Alexa Fluor 488 (1:400, Thermo Fisher Scientific, A-11001) diluted in blocking solution. Slides were washed again 3 times with PBS-Tween and mounted as described above.

### Genomic DNA extraction

For FFPE samples, 20 μm-thick sections were first dewaxed through 4 successive incubations in xylene for 3 min at 50 °C followed by successive washes with ethanol (100% - 95% - 70%). Genomic DNA was extracted from various cell lines and tissue samples (FFPE and frozen) by overnight digestion at 45 °C with 100 μg/ml of proteinase K and 50 μM CaCl_2_ in 600 μl lysis buffer (10 mM Tris-HCl, 10 mM EDTA, 1% SDS, pH 8.0) prior to DNA extraction with phenol-chloroform-isoamyl alcohol (25:24:1, Sigma-Aldrich) and chloroform (Sigma-Aldrich) and DNA precipitation with isopropanol and 0.3 M sodium acetate (pH 5.2). Genomic DNA was subsequently treated with 0.1 mg/ml RNAse A for 1 h at 37 °C, purified and precipitated again as described above.

### C-circle assay (CCA)

C-circle assay was performed as described in [24]. Briefly, up to 2 μg of RNA-free genomic DNA were digested with *Hinf*I/*Rsa*I and purified using phenol-chloroform extraction. Thirty ng of digested genomic DNA were resuspended in 10 μl of water and added to either 10 μl of reaction mix (4 μg/ml BSA, 0.1% Tween-20, 1 mM each dATP, dGTP and dTTP, 1x Phi29 Buffer and 7.5 U Phi29 DNA polymerase (NEB)) (+Φ29) or to 10 μl of the same mix lacking the Phi29 DNA polymerase (−Φ29). Samples were incubated at 30 °C for 8 h and then at 65 °C for 20 min. The amplification products were slot-blotted on a Hybond N+ nylon membrane (GE Healthcare). The membrane was pre-hybridized for 1 h at 42 °C in ULTRAhyb-Oligo hybridization buffer (Ambion) prior incubation with the radioactive telomeric probe in the same buffer for 16 h at 42 °C. The telomeric probe (*CCCTAA*)_4_ was prepared as follows: a 10 μM solution of telomeric sequence (Eurogentec, Belgium) was denatured for 5 min at 68 °C and 1 μl was used for radioactive labeling with 6 μl of [γ-^32^P] ATP (10 mCi/ml) (Perkin-Elmer) catalyzed by 10 U of T4 poly-nucleotide kinase (Sigma-Aldrich) in a 20 μl-reaction mix containing 1x PNK buffer; this mix was incubated for 20 min at 37 °C before inactivating the enzyme with 2 μl of EDTA (0.5 M, pH 8.0). Forty μl of 1x PNK buffer were then added to the radioactive probe before purification on a G-25 column. Post-hybridization, the membrane was washed first with Stringent wash buffer I for 20 min at RT and then with pre-warmed Stringent wash buffer II for 10 min at 42 °C (buffers provided in the Telo*TAGGG* kit, Sigma-Aldrich) and revealed using a Phosphorimager.

### Telomere restriction fragment (TRF) analysis

TRF analysis was performed as previously described [26]. Briefly, 10 μg of RNA-free genomic DNA were digested overnight with 20 U *Hinf*I and *Rsa*I and directly loaded on a 0.8% agarose gel. After 6 h of migration at 75 V, a depurination step was performed by soaking the gel 10 min in 0.25 M HCl followed by 30 min in denaturation buffer (0.5 M NaOH and 1.5 M NaCl) and again 30 min in neutralization buffer (0.5 M Tris-HCl, 3 M NaCl, 1 mM EDTA, pH 7.5). The gel was then placed in 20x SSC solution (3 M NaCl, 0.3 M sodium citrate, pH 7) and left overnight to transfer by capillarity on a Hybond N+ nylon membrane (GE Healthcare). The membrane was probed and revealed like described for CCA.

### RNA extraction and qRT-PCR

RNA was extracted from cells using ﻿TriPure Isolation Reagent (Sigma-Aldrich) followed by chloroform extraction and precipitation in isopropanol and 0.3 M sodium acetate (pH 5.2). The precipitate was treated with 2 U DNase I (TURBO™ DNase - Invitrogen) for 30 min at 37 °C followed by a second phenol-chloroform extraction. One μg of the purified RNA was reverse transcribed in cDNA using MMLV-RT (ThermoFisher Scientific) and random hexamers (ThermoFisher Scientific) according to the manufacturer’s instructions. qPCRs were performed on cDNA using ﻿KAPA SYBR FAST (Sigma-Aldrich) and the following primers (5′-3′): hTERT-F: *CGGAAGAGTGTCTGGAGCAA*; hTERT-R: *GGATGAAGCGGAGTCTGG*; hTR-F: *TTTGTCTAACCCTAACTAACTGAGAAG*; hTR-R: *TTGCTCTAGAATGAACGGTGGA*.

### Quantification and statistical analyses

﻿ImageJ was used to analyse confocal microscopy pictures, measure fluorescence intensity of ultra-bright telomeric signals and quantify slot-blots. Graphpad Prism 8.1.2 was used to generate graphs and for statistical analyses. Statistical analyses in this study were always performed using the unpaired nonparametric Mann-Whitney test. All *p* values ≤0.05 were considered as statistically significant.

## Supplementary Information

**Additional file 1.** Representative images of native FISH signals (red)in ALT^+^ and TEL^+^ cell lines. Scale bars: 5 μm.

## Data Availability

The datasets and materials generated during the current study are available from the corresponding author upon reasonable request.

## Abbreviations

ALT: Alternative Lengthening of Telomeres
ALT^+^: ALT-positive
APB: ALT-associated PML Body
CCA: C-circle Assay
ECTR: Extrachromosomal Telomeric Sequence
FFPE: Formalin-Fixed Paraffin-Embedded
FISH: Fluorescent in situ Hybridization
IF: Immunofluorescence
NSG: NOD Scid Gamma
PML: Promyelocytic Leukaemia
ss-TeloC: single-stranded C-rich telomeric DNA
TEL^+^: Telomerase-positive
TMM: Telomere Maintenance Mechanism
UBS: Ultra-Bright telomeric FISH Signals
