# Supplementary material for: Detection of alternative lengthening of telomeres mechanism on tumor sections
Source: Mol Biomed. 2021 Oct 20;2:32. doi: 10.1186/s43556-021-00055-y (PMC8607387; doi:10.1186/s43556-021-00055-y)
Supplement: Supplementary file 1 — Additional file 1. Representative images of native FISH signals (red)in ALT+ and TEL+ cell lines. Scale bars: 5 μm. [file 43556_2021_55_MOESM1_ESM.pdf]

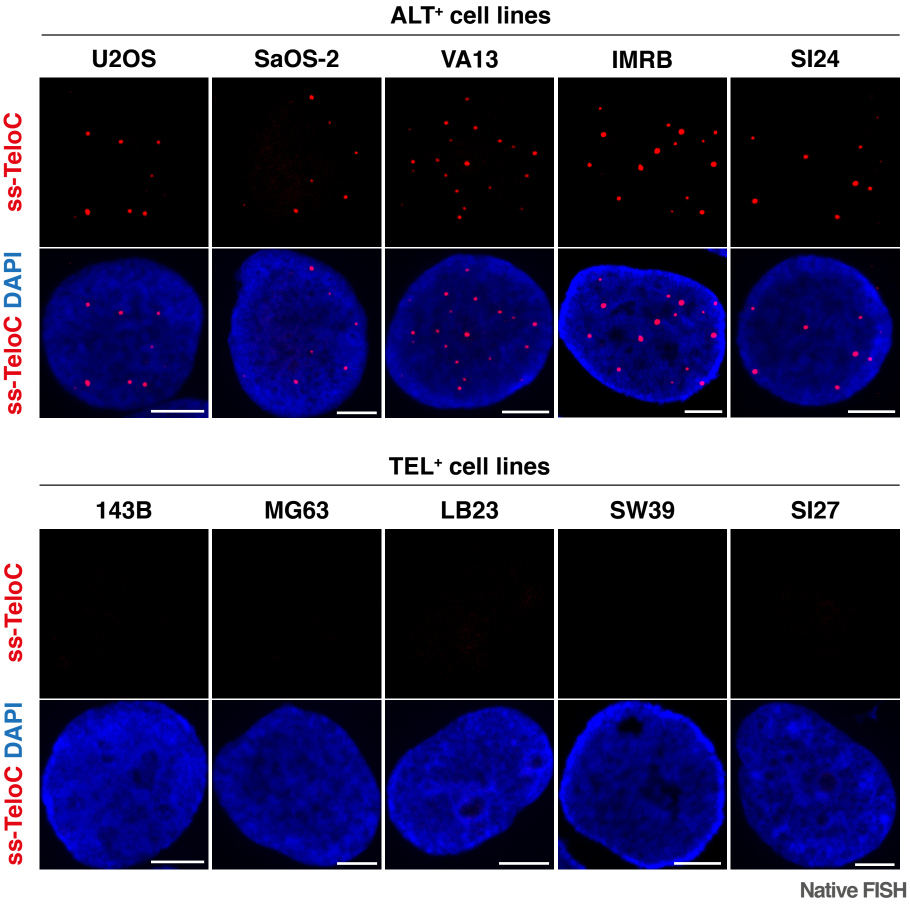

Online resource 1 Representative images of native FISH signals (red) in ALT^{+} and TEL^{+} cell lines. Scale bars: 5 μm
